# Relationship Between Macrocirculation and Microcirculation Monitored By Microdialysis During Septic Shock

**DOI:** 10.1186/2197-425X-3-S1-A522

**Published:** 2015-10-01

**Authors:** B Meddeb, Z Hajjej, H Gharsallah, B Trabelsi, I Labbene, M Ferjani

**Affiliations:** Military Hospital of Tunis, Tunis, Tunisia

## Introduction

Septic patients need a prompt normalization of macrohemodynamic parameters. Unfortunately, this optimization sometimes does not protect patients from organ failure development. in addition, there are data showing that microcirculatory dysfunction and Cellular metabolic alterations are associated with mortality and can fuel tissue distress leading to organ dysfunction [1].

## Objectives

The aim of our study was to explore the relationships between macrohemodynamic parameters and muscle tissue microdialysis-derived metabolites in critically ill septic patients.

## Methods

Prospective observational study included patients with septic shock admitted to a Tunisian medical -surgical ICU. Each patient was equipped with an arterial and a central venous catheter; patients were also monitored with a pulmonary artery catheter. Treatment for septic shock was standardized according to international recommendations. Microdialysis(MD) catheter was inserted in the femoral quadriceps. Dialysate samples were analyzed for glucose, pyruvate, lactate, and glycerol. the lactate/pyruvate (L/P) ratio was automatically calculated. Sampling was performed at baseline and every 6 h for 3 days. concomitantly with dialysate sampling, complete hemodynamic measurements were obtained. At the same time blood gases and serum lactate levels were measured. Relation between different parameters were assessed with Spearman’s rho test or the Pearson test p < 0.05 was considered significant.

## Results

We have included 30 patients with septic shock. a total of 390 measurements were performed. No significant correlation existed between mean arterial pressure(MAP) and tissue metabolites. MD lactate showed a significant, but weak correlation with cardiac index (rho= -0.128, p = 0.007). a negative weak correlations were found between systemic oxygen delivery index (DO_2_ I) and tissue lactate (rho= -0.234, p < 0.001), L/P ratio (rho= -0.142, p= 0.021) and tissue glucose (rho= -0.309, p < 0.001). No significant correlation existed between tissue L/P ratio and arterial oxygen partial pressure, mixed-venous oxygen saturation, or Systemic vascular resistance index. a significant positive correlations existed between tissue and blood lactate concentrations (rho= 0.298, P < 0.001) and between MD glucose and blood glucose (rho= 0.191, p < 0.001).

## Conclusions

Our results demonstrate a *decoupling* between macrocirculation and *microcirculation*. Conventional systemic hemodynamic- and oxygen-derived variables fail to detect such microcirculatory dysfunction and its response to the therapy in sepsis. Therefore, rather than limiting therapy to macrocirculatory targets alone, microcirculatory targets could be incorporated to potentially reduce mortality rates in these critically ill patients.

**Figure 1:**
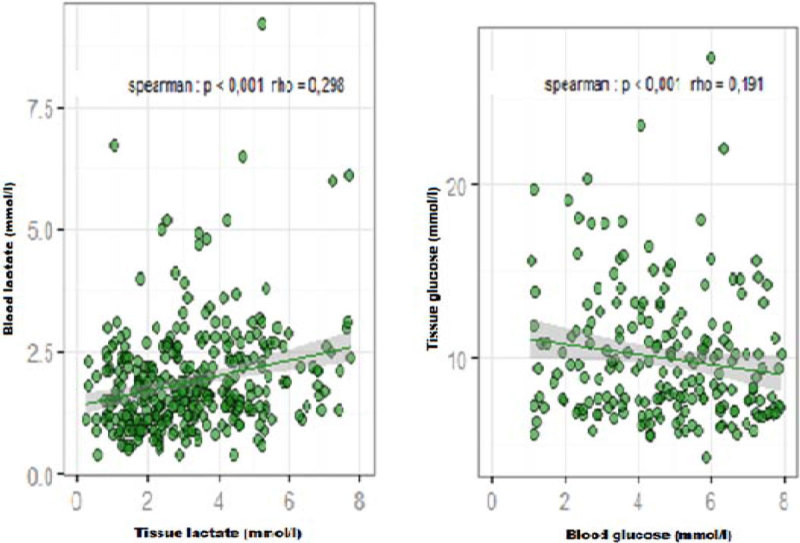
***Correlations between tissue and blood lactate.***
